# Epidemiological Link Between Post-traumatic Stress Disorder and Cardiovascular Disease: Evidence, Mechanisms, and Clinical Implications

**DOI:** 10.7759/cureus.100379

**Published:** 2025-12-29

**Authors:** Malik K Al-Ariki, Ivan Karpenko, Grigorii Esion, Anvar K Djumanov, Shirin Dadaev, Hasan Saghir, Daria Khorunzhaya, Zlata Kurant, Valeriia Gevorgian, Anastasiia V Badlaeva, Aleksey M Kireychev, Mohammad Ayad, Haya Darwich, Abubakar I. Sidik

**Affiliations:** 1 Department of Hospital Surgery, Peoples' Friendship University of Russia, Moscow, RUS; 2 Department of Cardiothoracic Surgery, A.A. Vishnevskiy Hospital, Moscow, RUS; 3 Department of Cardiovascular Surgery, A.A. Vishnevskiy Hospital, Moscow, RUS; 4 Department of Surgical Diseases No. 2, Tashkent Medical University, Tashkent, UZB; 5 Department of Cardiovascular Medicine, Novosibirsk State Medical University, Novosibirsk, RUS; 6 Department of Oncology, The P. A. Herzen Moscow Scientific Research Oncological Institute (MORI), Moscow, RUS; 7 Department of Cardiovascular Medicine, Volgograd State Medical University, Volgograd, RUS; 8 Department of Cardiovascular Medicine, Beirut Arab University, Beirut, LBN; 9 Department of Cardiovascular Surgery, Peoples' Friendship University of Russia, Moscow, RUS

**Keywords:** autonomic dysregulation, cardiovascular disease, epidemiology, inflammation, post-traumatic stress disorder (ptsd)

## Abstract

Post-traumatic stress disorder (PTSD) has emerged as a significant and independent risk factor for cardiovascular disease (CVD), yet its incorporation into routine cardiovascular prevention and care remains limited. This review synthesizes epidemiological evidence showing consistent associations between PTSD and increased incidence of hypertension, coronary artery disease, myocardial infarction, stroke, heart failure, and cardiovascular mortality across diverse populations, including veterans, civilians, women, and disaster-exposed cohorts. The review also highlights the bidirectional relationship between PTSD and CVD, since traumatic cardiovascular events such as myocardial infarction and cardiac arrest can precipitate persistent PTSD symptoms that negatively influence prognosis and recovery. Multiple biological and behavioral mechanisms are described, including autonomic dysregulation, activation of the hypothalamic-pituitary-adrenal (HPA) axis, systemic inflammation, endothelial dysfunction, metabolic disturbances, and maladaptive health behaviors. Clinical implications include gaps in current screening practices, the need for trauma-informed approaches in cardiovascular care, the impact of PTSD on treatment adherence and cardiac rehabilitation, and the potential role of PTSD-targeted interventions in reducing cardiovascular risk. Key limitations in the existing literature are identified, such as heterogeneity in diagnostic criteria, residual confounding from comorbid psychiatric conditions, and the underrepresentation of women and non-veteran populations. The review concludes by outlining future research priorities, with emphasis on mechanistic studies, longitudinal cohorts, clinical intervention trials, and the integration of social determinants of health. A deeper understanding of the complex relationship between PTSD and CVD is essential for advancing both prevention and clinical management in trauma-exposed individuals.

## Introduction and background

Cardiovascular diseases (CVDs) remain the leading cause of mortality worldwide, accounting for approximately 20.5 million deaths in 2021 and continuing to rise despite major advances in prevention and treatment [1]. The persistent global burden of CVD highlights the need to better understand non-traditional and understudied determinants of cardiovascular risk. One such factor gaining attention over the past two decades is post-traumatic stress disorder (PTSD), a psychiatric condition that may exert systemic physiological effects with long-term implications for cardiometabolic health.

PTSD is defined in the Diagnostic and Statistical Manual of Mental Disorders, Fifth Edition (DSM-5) as a trauma-related disorder characterized by intrusive re-experiencing, avoidance, negative alterations in cognition and mood, and heightened arousal following exposure to traumatic events involving actual or threatened death or serious injury [2]. Individuals with PTSD commonly experience nightmares, flashbacks, hypervigilance, and sleep disturbance, often resulting in substantial functional impairment and reduced quality of life [3].

Traumatic exposure is widespread, and epidemiological surveys suggest that lifetime PTSD prevalence ranges from 1% to 12% globally, with substantially higher rates observed in post-conflict settings [4]. Estimates indicate that 5% to 10% of trauma-exposed individuals develop PTSD, representing a considerable population at elevated risk for chronic disease [5]. Although historically conceptualized as a mental health disorder, accumulating evidence suggests that PTSD has far-reaching effects on physical health. Prior research has linked PTSD to obesity, metabolic syndrome, type 2 diabetes, and systemic inflammation, among other conditions [6,7]. These findings support a broader view of PTSD as a multisystem disorder mediated by chronic activation of stress pathways, autonomic imbalance, behavioral changes, and high rates of psychiatric comorbidity [8].

Within this broader context, epidemiological studies have increasingly identified PTSD as an independent risk factor for CVD. A meta-analysis of prospective cohort studies found that PTSD was associated with a 55% higher risk of incident coronary heart disease, even after adjustment for depression and other confounders [9]. In the Nurses’ Health Study II, trauma exposure combined with high PTSD symptom burden predicted significantly elevated risks of myocardial infarction and stroke among women over two decades of follow-up [10]. Large registry-based studies, including a nationwide sibling-controlled cohort from Sweden, have further demonstrated that stress-related disorders, including PTSD, are associated with increased risks of ischemic heart disease, stroke, heart failure, and cardiovascular mortality [11]. More recent studies from Europe and Asia have replicated these associations, providing evidence for increased risks of coronary artery disease, hemorrhagic stroke, and cardiometabolic multimorbidity across diverse populations [12,13]. Drawing on these findings, Edmondson and von Känel concluded that PTSD should be regarded as both a risk factor for incident CVD and a potential consequence of cardiac events, suggesting a bidirectional relationship between trauma and cardiovascular health [14].

Despite mounting evidence, PTSD is not routinely incorporated into cardiovascular risk assessment frameworks. Koenen and colleagues argue that PTSD predicts cardiometabolic disease risk to a degree comparable to several established risk factors, yet remains absent from clinical cardiovascular guidelines and algorithms [6]. Improved understanding of the pathways linking PTSD and CVD, and the populations most at risk, may enable more effective screening, risk stratification, and preventive intervention.

Given the growing body of research connecting PTSD with adverse cardiovascular outcomes, a focused synthesis of epidemiological evidence is needed. The purpose of this narrative review is to provide an integrated assessment of the epidemiological evidence connecting PTSD with CVD, to explore the biological and behavioral pathways that may account for this association, and to highlight the clinical and public health implications arising from this growing body of research. Through synthesis of findings across study designs and patient populations, the review aims to improve understanding of PTSD as a potential cardiovascular risk factor and to identify priorities for future research and clinical practice.

## Review

Approach to evidence synthesis

Because this article is designed as a narrative review, the approach to evidence gathering emphasized breadth, conceptual integration, and appraisal of key epidemiological contributions rather than exhaustive literature enumeration. The objective was to synthesize the most influential and methodologically robust studies examining the relationship between PTSD and CVD, with attention to diversity in study design, populations, and outcome measures.

The evidence reviewed was identified through targeted searches of major biomedical databases, including PubMed, Scopus, and Web of Science. Searches combined terms related to PTSD (for example, “post-traumatic stress,” “trauma-related disorders”) with cardiovascular outcomes (“cardiovascular disease,” “coronary heart disease,” “stroke,” “heart failure,” “cardiometabolic disease”). Reference lists of landmark articles, meta-analyses, cohort studies, and relevant reviews were hand-searched to identify additional sources. Priority was given to studies published in peer-reviewed journals, with a focus on prospective cohort designs, large registry-based analyses, and population-based epidemiological surveys. Although the majority of studies were drawn from the past two decades, earlier foundational work was also considered where relevant for historical context.

To ensure conceptual clarity and relevance, studies were included if they met the following general criteria: (1) PTSD was assessed using validated diagnostic instruments, structured clinical interviews, or established administrative definitions; (2) cardiovascular outcomes were objectively measured, clinically diagnosed, or extracted from validated registries; and (3) effect estimates were adjusted for key demographic and clinical confounders, such as age, sex, smoking, comorbid depression, and socioeconomic status (SES). Studies focusing solely on non-cardiovascular endpoints or those with insufficiently defined PTSD measures were excluded. Case reports, small uncontrolled clinical samples, and studies without clear temporal sequencing between PTSD exposure and cardiovascular outcomes were also not considered central to the synthesis.

The outcomes of interest ranged from incident cardiovascular events, such as myocardial infarction, stroke, heart failure, and sudden cardiac death, to broader endpoints, including cardiovascular mortality and composite cardiometabolic disease. Secondary outcomes included markers of subclinical cardiovascular pathology, such as coronary artery calcification, carotid intima-media thickness, endothelial dysfunction, and autonomic imbalance. Biomarkers reflective of inflammatory activity and neuroendocrine dysregulation were also reviewed, which informed the mechanistic understanding.

Although formal risk-of-bias scoring tools were not applied, the assessment of study quality followed accepted epidemiological principles. Greater weight was given to studies employing longitudinal designs, large and diverse populations, validated measures of PTSD and CVD, and comprehensive adjustment for confounding variables. The review also considered potential sources of bias, including residual confounding, misclassification of PTSD or cardiovascular outcomes, reverse causality, and differential healthcare utilization. Studies that employed advanced approaches to causal inference, such as sibling-controlled designs, marginal structural models, or instrumental variable strategies, were highlighted for their contributions to strengthening causal interpretation.

Epidemiological evidence linking PTSD and CVD

Cross-Sectional Studies

Cross-sectional studies provide an important early foundation for understanding the association between PTSD and cardiovascular risk, particularly through observations of elevated cardiometabolic abnormalities in individuals with PTSD. Although these studies cannot establish temporal or causal relationships, they consistently demonstrate that PTSD is associated with higher rates of hypertension, metabolic syndrome, and cardiovascular-related symptoms.

Multiple population-based cross-sectional analyses have shown that hypertension is more prevalent among adults with PTSD compared with those without the disorder. For example, a nationally representative study using data from the U.S. National Comorbidity Survey Replication found that individuals with PTSD exhibited significantly higher odds of hypertension, even after adjusting for sociodemographic characteristics and comorbid depression [15]. Similar findings have been reported among military personnel and veterans, where PTSD symptoms correlate strongly with elevated blood pressure and clinician-diagnosed hypertension [16].

PTSD has also been linked to an increased prevalence of metabolic syndrome, a cluster of cardiometabolic abnormalities that includes central obesity, dyslipidaemia, impaired glucose regulation, and elevated blood pressure. A systematic review and meta-analysis by Rosenbaum et al. found that individuals with PTSD had a 37% higher prevalence of metabolic syndrome compared with non-PTSD controls and significantly higher rates of abdominal obesity, hypertriglyceridaemia, and low high-density lipoprotein cholesterol [7]. These findings have since been replicated in both civilian and veteran samples, suggesting that metabolic dysregulation is a robust correlate of PTSD across diverse populations [17]. In addition to diagnosed cardiometabolic disorders, individuals with PTSD frequently report a higher burden of cardiac-related symptoms such as chest pain, palpitations, dyspnoea, and reduced exercise tolerance. Cross-sectional surveys of disaster survivors and trauma-exposed cohorts have shown elevated rates of self-reported cardiovascular symptoms among those with high PTSD symptom severity [18]. These symptoms may reflect underlying autonomic dysregulation, heightened sympathetic activity, or behavioural pathways such as physical inactivity and substance use.

Prospective Cohort Studies

Prospective cohort studies have been central to establishing the temporal association between PTSD and incident CVD. Unlike cross-sectional analyses, these designs follow individuals over time from the assessment of PTSD (or trauma exposure and symptoms) to the subsequent occurrence of cardiovascular events, thereby providing stronger evidence that PTSD precedes and potentially contributes to CVD risk.

Early longitudinal work in male veterans and community samples suggested that PTSD symptoms predict later coronary heart disease. In the Normative Aging Study, men with higher PTSD symptom levels at baseline had an increased risk of incident CHD over an average of nine years of follow-up, even after adjustment for traditional risk factors and depression [15]. Among Vietnam veterans, PTSD has similarly been associated with elevated risks of self-reported physician-diagnosed heart disease and hospitalization for cardiovascular conditions during follow-up [19].

Large cohort studies in veterans of more recent conflicts have strengthened this evidence. Analyses of U.S. Iraq and Afghanistan veterans receiving Veterans Health Administration care found that those with PTSD had a significantly higher incidence of myocardial infarction, stroke, and composite CVD outcomes compared with veterans without PTSD, with adjusted hazard ratios typically ranging from 1.3 to 1.8 [20,21]. These associations persisted after accounting for depression, substance use disorders, and a range of behavioural and clinical risk factors, suggesting an independent contribution of PTSD to cardiovascular risk.

Prospective data from civilian and general population cohorts show similar patterns. In the Nurses’ Health Study II, trauma exposure accompanied by high PTSD symptom burden predicted increased risks of incident myocardial infarction and stroke over two decades of follow-up in women, with multivariable-adjusted hazard ratios of approximately 1.5 to 1.6 compared with women without trauma exposure [10]. Importantly, part of this excess risk was explained by adverse health behaviours and cardiometabolic factors, underscoring their mediating role. In a large Swedish national cohort, individuals diagnosed with stress-related disorders, including PTSD, had markedly elevated risks of incident CVD compared with their unaffected siblings and population controls, particularly within the first year after diagnosis; although the relative risks attenuated over time, they remained significantly elevated throughout follow-up [11].

Several cohorts have reported dose-response relationships between PTSD symptom severity and cardiovascular outcomes. In the Nurses’ Health Study II, risk of CVD increased monotonically across categories of PTSD symptoms among trauma-exposed women, with the highest risk observed in those reporting the greatest symptom burden [10]. Similar gradients have been documented in veteran samples, where higher PTSD symptom scores predict greater incidence of CVD events and mortality [9,20]. These findings support the notion that more severe or persistent PTSD confers greater cardiovascular risk.

Sex- and age-stratified analyses indicate that the association between PTSD and CVD is evident in both men and women, although some studies suggest that women with PTSD may experience particularly elevated relative risks. In the Nurses’ Health Study II, the association between PTSD and CVD was demonstrated in a large cohort of middle-aged women [10], while veteran cohorts including both sexes have reported robust associations after adjustment for sex and age [20,21]. In the Swedish registry study, relative risks were generally similar across age groups, but absolute risks were higher among older individuals due to their greater baseline CVD incidence [11]. Overall, prospective studies across veterans, disaster survivors, and general population cohorts consistently show that PTSD and related stress disorders are associated with increased risk of incident cardiovascular events, with evidence of dose-response relationships and broadly similar effects across sex and age strata.

Case-Control Studies

Case-control studies provide important complementary evidence for the association between PTSD and acute cardiovascular events. By comparing individuals presenting with conditions such as acute coronary syndrome (ACS), stroke, or takotsubo cardiomyopathy to matched controls, these studies help clarify whether a history of PTSD or trauma-related symptoms is over-represented among patients experiencing acute CVD.

Several case-control investigations in cardiology settings have demonstrated a link between PTSD and ACS. In a study of myocardial infarction survivors, patients presenting with ACS were more likely to report a history of trauma exposure and significant PTSD symptoms than community controls, even after adjusting for conventional cardiovascular risk factors such as age, sex, smoking, and hypertension [22]. PTSD was also associated with poorer adherence to cardiac medications and increased risk of adverse outcomes following MI, suggesting that the disorder may influence both the development of ACS and its clinical course [22]. A separate meta-analytic case-control comparison found that individuals with ACS had substantially higher rates of prior PTSD than cardiac-healthy controls, reinforcing these findings [23].

Evidence regarding stroke shows a similar pattern. Case-control analyses have reported that individuals with ischaemic stroke are more likely to have preceding PTSD or screen positive for significant PTSD symptoms compared with stroke-free controls [24]. These associations were often independent of major vascular risk factors, supporting the hypothesis that PTSD may contribute to cerebrovascular vulnerability through pathways involving blood pressure dysregulation, endothelial dysfunction, or inflammation.

Takotsubo cardiomyopathy provides a particularly compelling context for case-control research because of its established association with emotional and physical stressors. Studies comparing takotsubo patients with ACS controls have found markedly higher rates of psychiatric disorders, including PTSD, among those with takotsubo syndrome [25]. One registry-based case-control study reported that nearly half of patients with takotsubo cardiomyopathy had a history of psychiatric illness, and PTSD was among the most frequently identified trauma-related diagnoses [25]. Another investigation found that takotsubo patients had significantly higher rates of psychiatric comorbidity than controls with typical ACS, further supporting an enhanced vulnerability linked to prior trauma and stress-related psychopathology [26].

Evidence in Specific Populations

Military and veteran populations compared with civilian populations: PTSD is highly prevalent among military personnel and veterans, making these groups central to understanding differential cardiovascular risk. Large-scale studies within the U.S. Veterans Health Administration have consistently shown that veterans with PTSD have significantly elevated risks of incident CVD, including myocardial infarction, stroke, and heart failure, compared with veterans without PTSD. Hazard ratios in these cohorts often range from 1.3 to 1.8 after adjusting for depression, substance use, and traditional cardiovascular risk factors [21,27]. The heightened physiological burden associated with chronic hyperarousal, combined with high rates of comorbid behavioural risk factors in veteran populations, may contribute to these elevated risks.

Civilian cohorts also demonstrate elevated CVD risk associated with PTSD, although absolute risk levels and contextual factors differ. In the Nurses’ Health Study II, a large U.S. civilian cohort, women with trauma exposure and high PTSD symptom burden showed a 50-60% increased risk of myocardial infarction and stroke over two decades of follow-up [10]. While the relative risks in civilians are broadly similar to those seen in military samples, the underlying trauma types, sociodemographic patterns, and comorbidities often differ. Civilian PTSD frequently arises from interpersonal violence, childhood trauma, or accidents, whereas combat exposure predominates in military settings.

Variations by trauma type (combat, interpersonal violence, and natural disasters): Trauma type appears to influence both PTSD presentation and associated cardiovascular risk. Combat trauma, which is often chronic and repeated, has been strongly linked to increased CVD in multiple veteran cohorts [19,27]. Interpersonal violence, which disproportionately affects women, is also associated with substantial cardiovascular burden. In the Nurses’ Health Study II, exposure to interpersonal violence coupled with high PTSD symptoms was particularly predictive of CVD events [10]. Trauma involving betrayal or interpersonal threat may trigger prolonged physiological stress responses that heighten cardiometabolic risk.

Natural and technological disasters represent another context in which PTSD and cardiovascular outcomes intersect. Studies of survivors of the September 11 attacks reported increased risk of physician-diagnosed heart disease among those with chronic, high-severity PTSD [28]. Similar findings have been observed in survivors of earthquakes, floods, and industrial accidents, who exhibit elevated rates of hypertension and cardiovascular symptoms when PTSD persists long after the event [29]. These disaster-related populations highlight how acute community-level trauma can produce long-term cardiovascular consequences.

Racial and ethnic differences linked to social determinants of health: Racial and ethnic disparities in PTSD-related CVD risk appear to reflect intersecting influences of trauma exposure, structural inequality, and access to care. In the U.S., Black and Hispanic populations experience higher rates of trauma exposure and often face cumulative socioeconomic disadvantage, which may amplify the cardiometabolic consequences of PTSD. Data from the World Trade Center Health Registry showed that Black and Hispanic survivors had higher rates of PTSD and subsequent cardiovascular morbidity compared with White survivors, in part due to disproportionate occupational exposures and limited post-disaster resources [12].

SES also shapes racial and ethnic variation in PTSD-CVD associations. A cohort study from South Korea found that individuals with PTSD had an elevated risk of CVD, and this association was significantly stronger among those with lower SES, suggesting that structural disadvantage modifies the impact of PTSD on cardiovascular health [12]. Similar patterns have been noted in U.S. veteran cohorts, where minority veterans with PTSD exhibit higher rates of hypertension, diabetes, and metabolic syndrome than White veterans with similar trauma histories [30]. These findings underscore the importance of considering social determinants, including income, neighbourhood environment, discrimination, and healthcare access, when evaluating cardiovascular risk in trauma-exposed populations. Table 1 summarises the principal research domains investigating the association between PTSD and CVD.

**Table 1:** Summary of Epidemiological Evidence Linking PTSD and CVD PTSD, Post-traumatic Stress Disorder; CVD, Cardiovascular Disease; LMICs, Low- and Middle-Income Countries

Evidence Category	Study Design Features	Key Findings	Strengths	Limitations
Cross-Sectional Studies [15-18]	Single time-point observational analyses comparing individuals with and without PTSD	Higher prevalence of hypertension, metabolic syndrome, cardiometabolic abnormalities, and cardiovascular symptoms among those with PTSD	Identifies robust associations and symptom patterns; useful for hypothesis generation	Cannot establish causality or temporal direction; vulnerable to confounding and self-report bias
Prospective Cohort Studies [9-11,15,19-21]	Longitudinal follow-up from PTSD exposure or symptoms to incident cardiovascular outcomes	PTSD predicts increased risk of coronary heart disease, myocardial infarction, stroke, heart failure, and cardiovascular mortality; dose-response relationships reported	Establishes temporal ordering; enables adjustment for covariates; strongest observational evidence	Residual confounding remains; limited mechanistic detail; PTSD definitions and outcome measures vary
Case-Control Studies [22-26]	Comparison of individuals with acute cardiovascular events (e.g., myocardial infarction, stroke) to matched controls	PTSD and trauma exposure are more common among acute coronary syndrome, stroke, and Takotsubo cardiomyopathy patients than in controls	Useful for rare or acute outcomes; highlights PTSD burden in clinical settings	Prone to recall and selection bias; temporal sequence less clear; smaller sample sizes
Evidence in Specific Populations [10,12,19,21,27,28-30]	Subgroup analyses including veterans, civilians, women, disaster-exposed cohorts, and racial/ethnic minorities	Elevated cardiovascular risk confirmed across groups; particularly strong relationships in veterans, women with interpersonal trauma, and disadvantaged populations	Demonstrates generalizability; highlights social determinants and trauma type differences	Many populations remain underrepresented (LMICs, rural communities); trauma heterogeneity complicates comparisons

Reverse Causality

Although most research highlights PTSD as a precursor to CVD, cardiovascular events themselves can act as psychologically traumatic stressors that precipitate clinically significant PTSD symptoms. Following ACS or myocardial infarction, pooled evidence indicates that approximately 10% to 12% of patients develop PTSD symptoms of clinical significance, with variation across assessment methods and populations [23,31]. Importantly, cardiac event-induced PTSD is not merely a transient reaction but a prognostically relevant condition. A meta-analysis reported that ACS-induced PTSD was associated with a nearly twofold higher risk of recurrent cardiac events or mortality compared with those without PTSD, independent of traditional risk factors [23].

Reverse causality has also been observed following other critical cardiovascular events. Among survivors of out-of-hospital cardiac arrest, systematic reviews have shown substantial prevalence of PTSD symptoms, often exceeding 15 to 20 percent during follow-up [32]. Likewise, ICU survivors, including those admitted for cardiac indications, exhibit persistent PTSD symptoms, with meta-analytic data indicating approximately one in five developing such symptoms after discharge [33,34]. To summarize the heterogeneity across populations and event types, the key findings from these studies are presented in Table 2.

**Table 2:** Evidence of Reverse Causality: Cardiovascular Events Leading to PTSD PTSD, Post-traumatic Stress Disorder; PTSS, Post-traumatic Stress Symptoms; ACS, Acute Coronary Syndrome; MI, Myocardial Infarction; CABG, Coronary Artery Bypass Grafting; CHD, Coronary Heart Disease; SCAD, Spontaneous Coronary Artery Dissection; ICD, Implantable Cardioverter-Defibrillator; VAD, Ventricular Assist Device; LVAD, Left Ventricular Assist Device; CHD (pediatric), Congenital Heart Disease; TIA, Transient Ischemic Attack; OHCA, Out-of-Hospital Cardiac Arrest; DSM, Diagnostic and Statistical Manual of Mental Disorders; SCID, Structured Clinical Interview for DSM; IES-R, Impact of Event Scale–Revised; PCL-5, PTSD Checklist for DSM-5; ICU, Intensive Care Unit; SD, Standard Deviation; N, Sample Size.

Study	Cardiovascular Event	Sample/Population	PTSD or PTSS Findings	Assessment Method & Timing
Edmondson et al., 2013 [35]	Stroke/TIA	9 studies (N = 1,138)	PTSD prevalence 23% within 1 year, 11% beyond 1 year	Structured PTSD assessments
Edmondson et al., 2012 [9]	ACS	CHD cohorts	ACS-induced PTSD doubled the risk of recurrent events & mortality	Validated PTSD scales; prospective follow-up
Johnson et al., 2020 [36]	SCAD	512 SCAD survivors	28% had mild–severe PTSD symptoms	PTSD Diagnostic Scale for DSM-5
Ladwig et al., 2008 [37]	ICD implantation	147 ICD patients	Upper-quartile PTSD → 3.45× mortality risk	Impact of Event Scale–Revised
Meentken et al., 2017 [38]	Pediatric CHD surgery	Pediatric surgical cohorts	PTSD prevalence 12–31%	Various pediatric PTSD tools
Presciutti et al., 2021 [39]	Cardiac arrest	169 survivors; 52 caregivers	PTSD: 24.9% survivors; 34.6% caregivers	PTSD Checklist–5
Rawashdeh et al., 2020 [40]	CABG surgery	148 post-CABG patients	PTSD prevalence 20.3%; predicted by ICU stay and complications	Impact of Event Scale–Revised
Righy et al., 2019 [33]	ICU critical illness	Meta-analysis (48 studies)	Pooled PTSD prevalence 19.8% (3.7–43.7%)	Validated PTSD instruments
Sears et al., 2011 [41]	ICD implantation	Review of ICD cohorts	PTSD 20–36%, up to 48% over time	PTSD interviews & questionnaires
Singh et al., 2017 [42]	MI and CABG	Review of MI/CABG literature	MI-PTSD 12–30%; CABG-PTSD 8–18%	Structured interviews & questionnaires
Weerahandi et al., 2017 [43]	VAD implantation	87 VAD patients	No PTSD cases detected	SCID
Yaow et al., 2022 [32]	Out-of-hospital cardiac arrest	Meta-analysis (13 studies; N=186,160)	PTSD prevalence 20%	Validated PTSD scales

Several interconnected mechanisms underlie this reverse causal pathway. Physiological stress responses during acute cardiac events trigger excessive sympathetic activation, catecholamine release, and systemic inflammation, contributing to both psychological trauma and recurrent cardiac dysfunction. Dysregulation of the hypothalamic-pituitary-adrenal (HPA) axis and maladaptive behavioural changes such as reduced physical activity, avoidance of medical care, and poor medication adherence further compound cardiovascular risk [8,14,44]. The Mind Your Heart study demonstrated that patients with PTSD following cardiovascular events were significantly less likely to adhere to prescribed cardioprotective medications, reinforcing behavioural mechanisms of disease recurrence [44].

Subclinical Cardiovascular Changes

Research increasingly suggests that PTSD is associated with several subclinical cardiovascular abnormalities that may help explain how psychological trauma contributes to the development of CVD. These intermediate markers include coronary artery calcification (CAC), endothelial dysfunction, alterations in autonomic regulation, inflammatory activation, and early changes in vascular stiffness.

CAC: CAC, an established marker of subclinical atherosclerosis, has been examined in selected clinical cohorts. In a study of United States veterans who underwent coronary computed tomography, individuals with PTSD had a higher prevalence of coronary atherosclerosis and calcification compared with those without PTSD [45]. Although these findings suggest possible accelerated atherosclerosis, the evidence base remains limited, and larger longitudinal studies specifically focused on CAC in PTSD populations are needed.

Endothelial dysfunction: Endothelial dysfunction is one of the most consistently documented subclinical abnormalities in PTSD. In a cohort of trauma-exposed adults, those with PTSD demonstrated significantly impaired endothelial function, measured through flow-mediated dilation, compared with trauma-exposed controls without PTSD [46]. Longitudinal research in a large cohort of women showed that chronic PTSD symptoms were associated with elevated biomarkers of endothelial activation, including ICAM-1 and VCAM-1, over more than a decade of follow-up [47]. More recent work in trauma-exposed young women also found that greater PTSD symptom severity was associated with impaired microvascular endothelial function as measured by the reactive hyperaemia index [48].

Heart rate variability (HRV) alterations: Autonomic dysregulation is another frequently observed physiological feature of PTSD. Reduced heart rate variability, indicating lower parasympathetic activity and higher sympathetic activation, has been demonstrated across multiple studies. In a large cohort of veterans, individuals with PTSD exhibited significantly lower HRV and indicators of heightened autonomic dysregulation compared with controls [49]. These findings are clinically important because low HRV is associated with arrhythmias, sudden cardiac death, and increased risk of cardiovascular events.

Inflammatory markers: PTSD is also consistently linked to a pro-inflammatory biological profile. Longitudinal data from a large cohort of women showed that the onset of PTSD symptoms was followed by elevated inflammatory markers, including C-reactive protein (CRP), tumour necrosis factor (TNF) receptor II, and other circulating cytokines, even after accounting for relevant behavioural and medical risk factors [47]. Additional studies have reported similar increases in inflammatory cytokine production and immune dysregulation in PTSD, supporting the presence of chronic low-grade inflammation in this population [49].

Arterial stiffness and vascular reactivity: Emerging evidence also suggests that PTSD is associated with increased arterial stiffness and impaired vascular reactivity. In a study of trauma-exposed young women, PTSD symptom severity and poor sleep efficiency were associated with greater arterial stiffness as measured by pulse-wave velocity, along with impaired microvascular reactivity [48]. These early indicators of vascular dysfunction may form part of the pathway through which PTSD increases long-term cardiovascular risk.

Biological and behavioral mechanisms

Autonomic Nervous System Dysregulation

Autonomic nervous system dysregulation is one of the most consistently observed physiological mechanisms linking PTSD to elevated cardiovascular risk. Individuals with PTSD frequently exhibit evidence of heightened sympathetic activation and reduced parasympathetic tone, which together contribute to a chronic state of autonomic imbalance. This imbalance can increase cardiovascular workload, promote vascular inflammation, and impair the body's ability to regulate stress responses.

A substantial body of research has documented elevated sympathetic activity in PTSD. Individuals with PTSD show increased resting heart rate, elevated catecholamine levels, and exaggerated cardiovascular responses to stress compared with trauma-exposed or non-exposed controls [50]. These heightened responses reflect persistent activation of the sympathetic nervous system, which may accelerate cardiovascular wear and tear over time.

Reductions in HRV represent one of the clearest indicators of autonomic dysregulation in PTSD. HRV is a marker of parasympathetic function and overall autonomic flexibility. Multiple studies have shown that people with PTSD have significantly lower HRV than controls, including reductions in both time-domain measures, such as root mean square of successive differences, and frequency-domain measures such as high-frequency HRV [51]. These reductions in HRV reflect diminished vagal activity and impaired capacity to recover from stress.

A large study of United States veterans found that PTSD was associated with pronounced autonomic dysregulation, including lower HRV and exaggerated skin conductance responses, even after accounting for demographic factors, smoking, and medical comorbidities [52]. These findings reinforce the evidence that autonomic imbalance is not solely a correlate of acute distress but represents a chronic physiological state in individuals with PTSD.

Autonomic dysregulation in PTSD is clinically significant because reduced HRV and increased sympathetic tone are well-established predictors of incident cardiovascular events, arrhythmias, sudden cardiac death, and all-cause mortality. These autonomic alterations may therefore represent a key biological mechanism by which PTSD increases vulnerability to coronary artery disease and other cardiovascular conditions.

HPA Axis Abnormalities

Dysregulation of the HPA axis is a central biological mechanism through which PTSD may increase cardiometabolic risk. The HPA axis governs the secretion of cortisol, a key hormone involved in stress regulation, metabolism, immune function, and cardiovascular homeostasis. Numerous studies have found that individuals with PTSD exhibit abnormalities in basal cortisol levels, diurnal cortisol rhythms, and cortisol reactivity to stress, all of which may contribute to adverse cardiovascular outcomes.

A widely replicated finding is that individuals with chronic PTSD often display lower basal cortisol levels and blunted cortisol responses to acute stress. A meta-analysis of HPA axis activity in PTSD found consistent evidence of reduced morning cortisol concentrations and heightened sensitivity of the glucocorticoid receptor system, suggesting altered negative feedback regulation [53]. These cortisol patterns contrast with the elevations typically observed in acute stress and may reflect long-term adaptation of the HPA axis following trauma exposure.

Blunted or dysregulated cortisol activity can have important cardiometabolic consequences. Low basal cortisol and disrupted diurnal rhythms have been linked to increased systemic inflammation, endothelial dysfunction, and sympathetic nervous system activation, all of which are established risk pathways for CVD. In a study of Bosnian war refugees with PTSD, individuals demonstrated lower cortisol levels accompanied by significantly increased production of inflammatory cytokines in response to stress challenges [54]. This pattern illustrates how HPA axis hypoactivity may fail to restrain inflammatory responses, thereby promoting vascular injury and atherogenesis.

PTSD has also been associated with altered diurnal cortisol slopes, including flatter cortisol decline across the day. In the Multi-Ethnic Study of Atherosclerosis, flatter cortisol slopes were associated with increased prevalence of hypertension, diabetes, and subclinical CVD [55]. Although this study did not focus exclusively on PTSD, the pathways it describes are relevant because similar cortisol abnormalities have been observed in trauma-exposed populations, suggesting shared endocrine mechanisms underlying cardiometabolic vulnerability.

Inflammation and Immune Activation

Chronic inflammation is a key biological mechanism that may help explain the elevated cardiovascular risk observed in individuals with PTSD. Numerous studies indicate that people with PTSD exhibit increased circulating levels of inflammatory markers, including CRP, interleukin-6 (IL-6), and TNF alpha (TNF-α). These inflammatory abnormalities are clinically significant because elevated levels of these biomarkers have been linked to the development of atherosclerosis, endothelial dysfunction, and future CVD.

Several studies have reported higher CRP concentrations among individuals with PTSD compared with trauma-exposed or non-exposed controls. In the Nurses' Health Study II, chronic PTSD symptoms were associated with significantly higher CRP levels, even after controlling for body mass index, smoking, and depressive symptoms [47]. Similar findings have been observed in veteran samples, where CRP elevations were related to both PTSD diagnosis and symptom severity [52].

Inflammatory cytokines such as IL-6 and TNF-α are also consistently elevated in PTSD. In a controlled study of PTSD patients, individuals exhibited significantly higher levels of IL-6 and TNF-α compared with matched healthy controls, suggesting upregulated inflammatory signalling pathways [56]. Another large study reported that PTSD was associated with greater inflammatory cytokine responses to laboratory stressors, indicating both heightened baseline inflammation and exaggerated inflammatory reactivity [52].

These inflammatory abnormalities have direct relevance for CVD risk. Elevated CRP and IL-6 contribute to endothelial activation, oxidative stress, and plaque formation, while TNF-α has been implicated in insulin resistance, vascular remodelling, and progression of atherosclerotic lesions. Longitudinal evidence further suggests that individuals with persistent PTSD show continuing increases in inflammatory biomarkers over time, which may accelerate cardiometabolic deterioration [47].

Health Behaviors

Health behaviours represent an important pathway through which PTSD may increase CVD risk. Individuals with PTSD frequently engage in behaviours that negatively affect cardiometabolic health, including smoking, unhealthy diet, physical inactivity, alcohol misuse, and poor sleep. These behaviours can amplify biological vulnerabilities such as inflammation, autonomic dysregulation, and metabolic dysfunction, thereby accelerating the development of CVD.

Smoking is one of the most consistently reported maladaptive behaviours among individuals with PTSD. Epidemiological studies show that people with PTSD are more likely to smoke, smoke more heavily, and have lower cessation rates than those without PTSD [57]. Smoking contributes directly to endothelial injury, oxidative stress, and atherosclerosis, which increases the risk of MI and stroke.

Unhealthy dietary patterns are also more common among individuals with PTSD. Studies have reported higher intake of processed foods, sugar-sweetened beverages, and saturated fats in trauma-exposed populations, along with lower consumption of fruits and vegetables [58]. Poor dietary quality contributes to obesity, insulin resistance, and dyslipidemia, all of which elevate cardiovascular risk.

Physical inactivity is another common behaviour in individuals with PTSD. Symptoms such as avoidance, low motivation, and chronic fatigue can limit participation in regular exercise. Findings from large prospective cohorts indicate that trauma exposure and PTSD symptoms are associated with reduced physical activity and increased sedentary behaviour, both of which negatively affect cardiovascular health [59].

Alcohol misuse is widely documented in PTSD and represents a significant behavioural mechanism linking PTSD to CVD. Individuals with PTSD are at increased risk for hazardous drinking, binge drinking, and alcohol use disorders [5]. Excessive alcohol consumption contributes to hypertension, cardiomyopathy, arrhythmias, and metabolic dysfunction.

**Figure 1:**
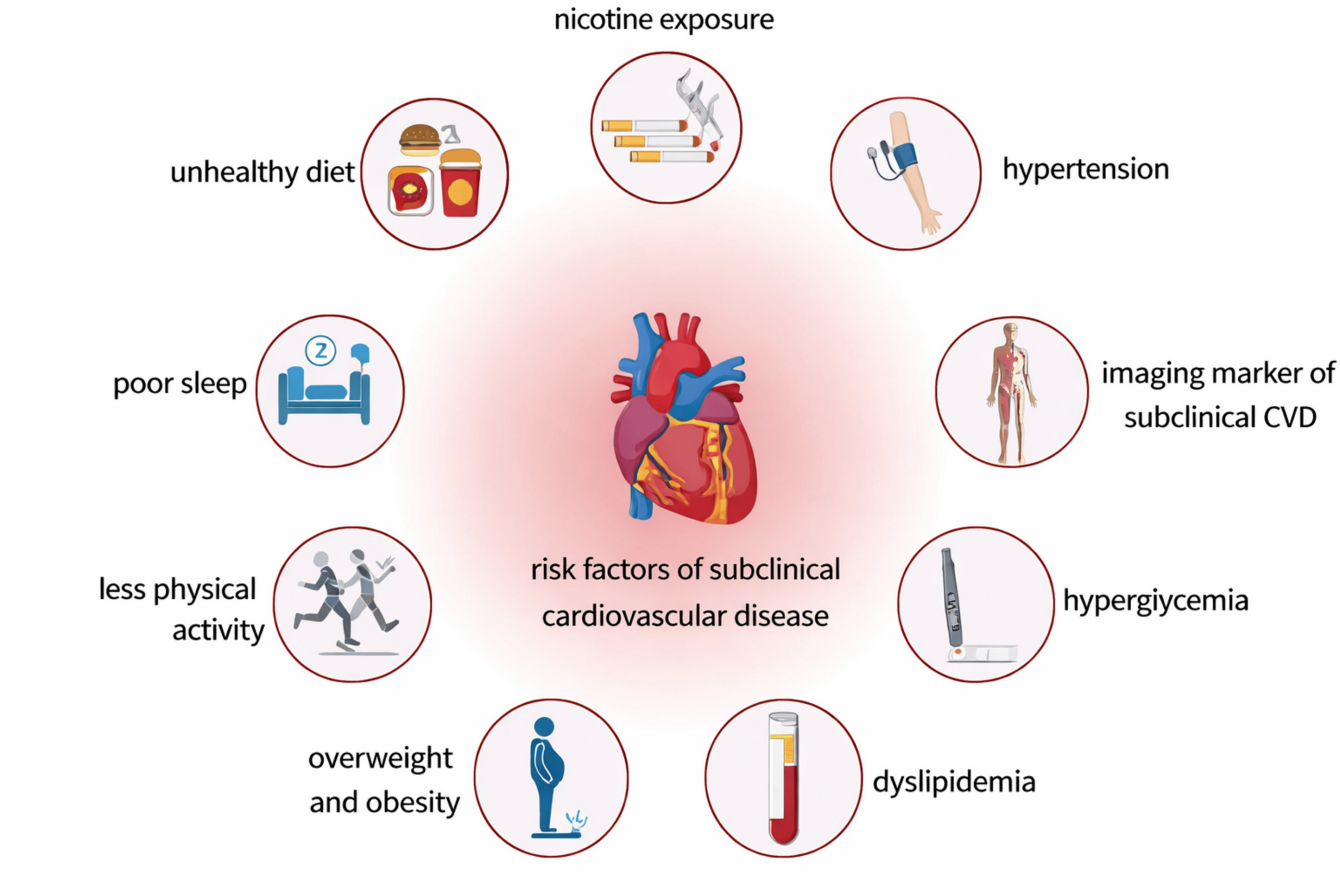
Behavioral and Cardiometabolic Risk Factors Linking Post-traumatic Stress Disorder to Subclinical Cardiovascular Disease (CVD). Individuals with post-traumatic stress disorder frequently exhibit adverse health behaviors and metabolic abnormalities, including nicotine exposure, unhealthy dietary patterns, physical inactivity, poor sleep, overweight and obesity, hypertension, hyperglycemia, and dyslipidemia. These interrelated risk factors contribute to the development of subclinical CVD, detectable through imaging and other early markers of vascular and cardiometabolic dysfunction. This is an original image created by the authors.

Poor sleep is a hallmark of PTSD and a key behavioural factor in cardiovascular risk. Insomnia, nightmares, and fragmented sleep are common and have been linked to hypertension, impaired glucose metabolism, and increased inflammatory markers. A growing body of research shows that individuals with PTSD exhibit shorter sleep duration, lower sleep efficiency, and more frequent sleep disturbances than non-PTSD controls [60]. These sleep abnormalities contribute to both sympathetic nervous system activation and metabolic dysregulation. PTSD is associated with a constellation of adverse health behaviors and metabolic abnormalities that converge to promote subclinical CVD (Figure 1).

Comorbid Mental Health Conditions

Comorbid mental health conditions, particularly depression and anxiety disorders, play an important role in modifying and potentially mediating the association between PTSD and CVD. PTSD frequently co-occurs with depressive and anxiety symptoms, and these comorbidities can amplify both biological and behavioural risk pathways that contribute to cardiovascular vulnerability.

Depression is one of the most common comorbidities in PTSD and is itself an established risk factor for CVD. Large epidemiological studies have shown that individuals with both PTSD and depression have significantly higher risks of incident myocardial infarction and stroke compared with those with PTSD alone [9]. Depression can worsen behavioural risk factors such as physical inactivity, poor diet, and smoking, while also contributing to physiological processes including autonomic imbalance, inflammation, and endothelial dysfunction. These combined effects may heighten cardiovascular risk beyond the contribution of PTSD alone.

Anxiety disorders are also frequently observed alongside PTSD and may further modify cardiovascular outcomes. Anxiety is associated with elevated sympathetic nervous system activity, increased circulating catecholamines, and heightened cardiovascular reactivity. A meta-analysis examining anxiety disorders found that these conditions were linked to increased risk of coronary heart disease and cardiovascular mortality, even after adjusting for traditional risk factors [61]. When anxiety co-occurs with PTSD, these physiological effects may be exacerbated, leading to greater cardiovascular strain.

The interaction of PTSD with depression and anxiety may operate through both direct biological mechanisms and indirect behavioural pathways. For example, individuals with comorbid PTSD and depression exhibit higher inflammatory marker levels, reduced heart rate variability, and more severe endothelial dysfunction than those with either condition alone [62]. These synergistic effects suggest that comorbidity is more than an additive risk factor and may represent a distinct psychophysiological profile associated with greater cardiovascular vulnerability.

**Figure 2:**
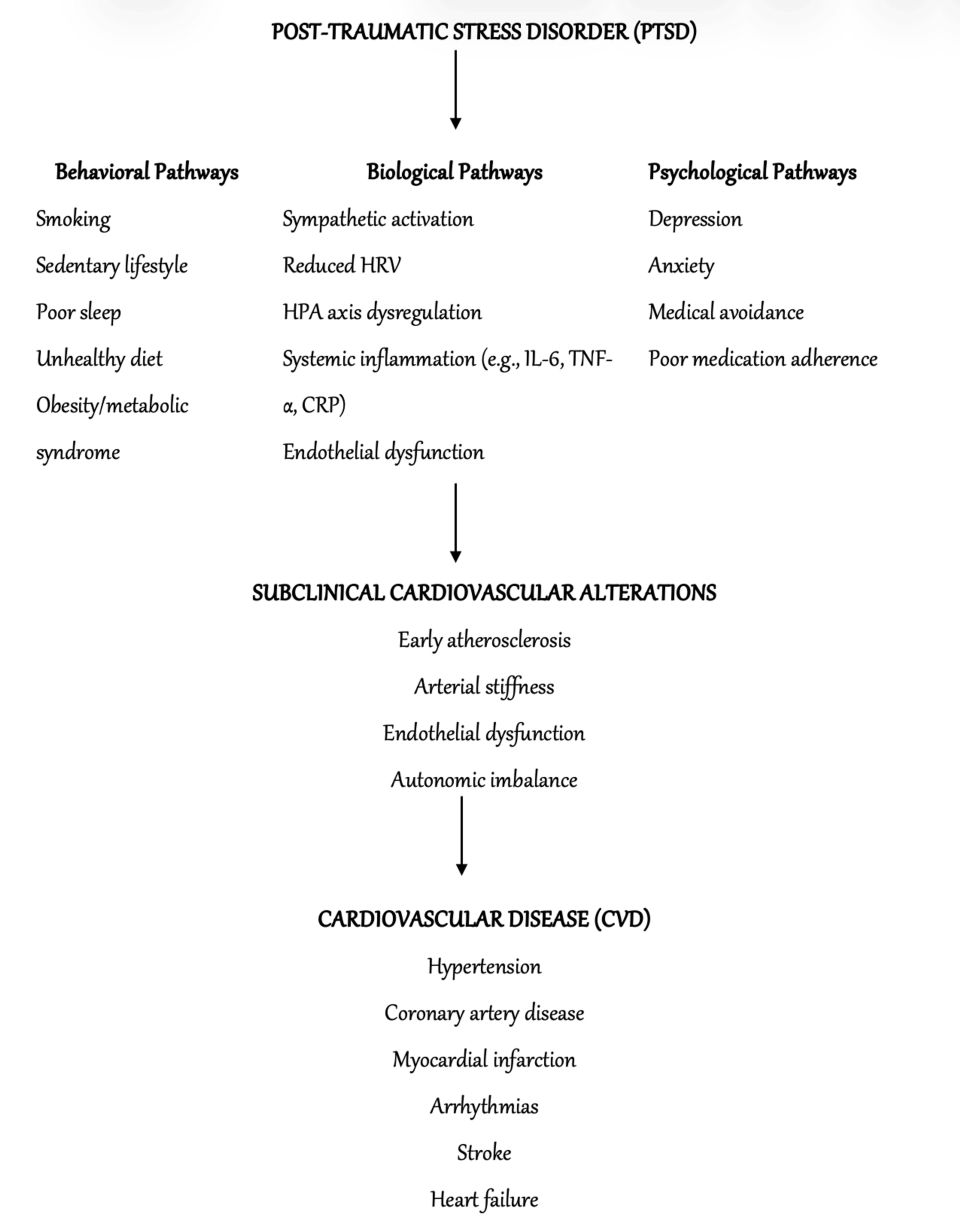
Mechanisms Linking PTSD to Cardiovascular Disease (CVD) Summary of the key biological and behavioural pathways through which PTSD increases cardiovascular risk, including autonomic imbalance, HPA axis dysregulation, chronic inflammation, endothelial dysfunction, and adverse health behaviours that collectively promote atherosclerosis and cardiometabolic disease. PTSD, Post-traumatic Stress Disorder; HRV, Heart Rate Variability; HPA, Hypothalamic-Pituitary-Adrenal; IL-6, interleukin-6; TNF-α; Tumour Necrosis Factor Alpha; CRP: C-Reactive Protein This is an original image created by the authors.

In addition, comorbid depression and anxiety can impair adherence to medical treatments, reduce engagement in preventive health behaviours, and increase the likelihood of delayed care seeking. These behavioural effects further compound cardiovascular risk in individuals with PTSD. These interrelated behavioural, biological, and psychological mechanisms collectively illustrate how PTSD contributes to the development of CVD, as summarised in Figure 2.

Clinical implications

Importance of Screening for PTSD in Cardiac Patients

The growing evidence linking PTSD to elevated CVD risk highlights the need for systematic screening for PTSD in cardiac care settings. Studies of patients hospitalised with ACS have shown that a substantial proportion meet criteria for PTSD, and these patients often experience poorer adherence to treatment, reduced quality of life, and higher rates of recurrent cardiovascular events [23]. Early identification of PTSD symptoms in cardiac patients could facilitate appropriate referral to mental health services and help mitigate the adverse effects on cardiovascular prognosis.

Integrating Mental Health Services in Cardiology and Primary Care

Integrating mental health services into cardiology and primary care settings has been proposed as a key strategy to address comorbid PTSD and CVD. Collaborative care models, which include coordinated management by primary care providers, cardiologists, and mental health professionals, have demonstrated effectiveness in improving both mental health outcomes and cardiometabolic indicators in patients with depression and anxiety [63]. Although similar models focused specifically on PTSD remain limited, the structure of collaborative care is well-suited to managing the complex interplay of behavioural and biological risk factors seen in PTSD.

Importance of Trauma-Informed Care

Trauma-informed care is increasingly recognised as an essential component of cardiovascular prevention and treatment in individuals with PTSD. Trauma-informed approaches emphasise safety, trust, patient empowerment, and sensitivity to trauma histories. Research suggests that patients with PTSD often report negative healthcare experiences related to perceived judgment or lack of understanding, which can reduce engagement in care and adherence to treatment [64]. Implementing trauma-informed communication and clinical practices may therefore improve the therapeutic relationship and increase patient participation in cardiovascular risk reduction strategies.

Potential Role of PTSD Treatment in Modifying CVD Risk

There is growing interest in whether treatment of PTSD may help reduce cardiovascular risk. Evidence suggests that evidence-based psychotherapies for PTSD, such as cognitive behavioural therapy and prolonged exposure therapy, can reduce symptoms of hyperarousal and improve behavioural risk factors that contribute to CVD. Observational studies in veterans have found that effective PTSD treatment is associated with lower rates of hypertension and improved metabolic parameters [21].

Pharmacological treatments, particularly selective serotonin reuptake inhibitors, have been shown to reduce PTSD symptoms and may indirectly influence cardiovascular health through reductions in inflammation, autonomic dysregulation, and health-risk behaviours [64]. In addition, structured exercise programs have demonstrated beneficial effects on both PTSD symptoms and cardiometabolic health, including improvements in blood pressure, insulin sensitivity, and overall physical functioning [65].

Public health and policy implications

Burden of PTSD in Vulnerable Populations

PTSD disproportionately affects populations exposed to chronic adversity, violence, or disaster, which results in an unequal distribution of CVD risk. Epidemiological surveys show higher rates of PTSD among individuals with low SES, racial and ethnic minority groups, refugees, and survivors of interpersonal violence [4]. These populations often experience limited access to healthcare, greater exposure to environmental stressors, and higher rates of comorbid mental health and cardiometabolic conditions. As a result, PTSD may amplify existing health disparities and contribute to disproportionate CVD burden.

Need for Longitudinal Surveillance and Large-Scale Cohort Studies

Despite growing recognition of the relationship between PTSD and CVD, longitudinal population-based studies remain relatively limited. Large-scale cohort studies are essential for clarifying temporal relationships, evaluating causal pathways, and identifying factors that modify cardiovascular risk over time. The Swedish national cohort study on stress-related disorders represents a model for high-quality epidemiological surveillance, demonstrating increased risks of ischaemic heart disease, stroke, and cardiovascular mortality among individuals with PTSD compared with their unaffected siblings [11]. Expanding such efforts across diverse populations and healthcare systems would inform targeted prevention strategies and more precise risk prediction models.

Implications for Veterans' Healthcare Policy

Veterans represent a population with particularly high rates of PTSD, and policy efforts within veterans' healthcare systems have increasing relevance for cardiovascular risk reduction. Studies from the United States Veterans Health Administration show that veterans with PTSD have a significantly higher incidence of hypertension, diabetes, and coronary artery disease than veterans without PTSD [27]. These findings highlight the need for integrated mental health and cardiovascular screening within veterans' health services. Policy initiatives that support early PTSD detection, trauma-informed care training, and coordinated management of mental and physical health could reduce long-term CVD burden in this group.

Strategies for Primary and Secondary Prevention

Primary and secondary prevention strategies are critical for mitigating cardiovascular risk in individuals with PTSD. Primary prevention efforts should include screening for PTSD in primary care and emergency settings following trauma exposure, coupled with timely referral to evidence-based psychological treatments. Secondary prevention should focus on integrating PTSD management with cardiovascular risk reduction programs. This could include behavioural interventions that target smoking cessation, improved diet, increased physical activity, and sleep hygiene, all of which have shown benefits in PTSD populations [65]. Public health policies that increase access to mental health services, promote stable housing, reduce exposure to violence, and address socioeconomic inequities may also help reduce the onset and persistence of PTSD and its downstream cardiovascular effects.

Limitations of epidemiological evidence

Heterogeneity in PTSD assessment (PTSD Checklist for DSM-5 (PCL-5) vs. Clinician-Administered PTSD Scale (CAPS))

A significant limitation in the current literature examining the relationship between PTSD and CVD is the heterogeneity in PTSD assessment methods. Studies differ widely in how PTSD is diagnosed and quantified, which affects comparability, reproducibility, and the strength of pooled evidence. The two most commonly used instruments are the PTSD Checklist (PCL, including PCL-5) and the CAPS. While both tools are validated, they measure symptoms using different methodologies, thresholds, and administration contexts, leading to inconsistencies across epidemiological and clinical studies [66,67].

The PCL-5 is a self-report measure based on DSM-5 criteria, widely used in large-scale epidemiological studies due to its simplicity and cost-effectiveness. However, it relies on subjective symptom reporting, which may be influenced by recall bias, comorbid depression, and somatic symptom overlap with cardiovascular conditions [68]. By contrast, the CAPS is a structured clinical interview regarded as the gold standard for PTSD diagnosis, offering greater diagnostic precision and consistency. Yet, its administration is time-consuming and resource-intensive, limiting its use in population-based studies [69].

This variability in diagnostic instruments has direct implications for estimating PTSD prevalence and its association with CVD. Studies using self-reported measures such as the PCL-5 tend to report higher PTSD prevalence and stronger associations with cardiovascular outcomes than those using clinician-administered interviews like CAPS [70]. Furthermore, differences in cutoff scores, reference time frames (for example, past month versus lifetime), and symptom cluster weighting contribute to inconsistencies in effect sizes and risk estimates across studies [71].

Confounding by Trauma Type and Comorbidities

Another major limitation in the current evidence linking PTSD and CVD is confounding by trauma type and psychiatric or medical comorbidities. PTSD is a heterogeneous condition that can arise from diverse traumatic experiences such as combat exposure, sexual assault, natural disasters, or medical trauma, and these trauma types differ significantly in their biological, behavioural, and psychosocial consequences [72]. For instance, combat-related PTSD is frequently associated with chronic hyperarousal and high levels of sympathetic activation, whereas interpersonal trauma such as sexual assault or childhood abuse often leads to heightened inflammation, metabolic dysregulation, and altered HPA axis function [10]. These trauma-specific patterns may contribute differently to cardiovascular risk.

Additionally, PTSD rarely occurs in isolation. High rates of comorbid depression, anxiety, and substance use disorders complicate the interpretation of studies on PTSD and CVD [9]. Depression and anxiety independently increase the risk of hypertension, coronary artery disease, and myocardial infarction through mechanisms such as systemic inflammation, autonomic dysregulation, and unhealthy lifestyle behaviours [21]. Substance use disorders, especially tobacco, alcohol, and stimulant use, are also highly prevalent in PTSD populations and can directly contribute to atherosclerosis, endothelial dysfunction, and arrhythmias [73].

Failure to adequately adjust for these comorbidities can lead to overestimation of the direct effect of PTSD on cardiovascular outcomes. Conversely, in some studies, over-adjustment for variables that are mechanistically linked to PTSD, such as depression or smoking, may underestimate its true causal contribution. Trauma severity, chronicity, and cumulative exposure, for example, repeated deployment in military personnel, also influence CVD risk but are inconsistently measured across studies [74].

Underrepresentation of Women and Non-veteran Populations

A major methodological limitation in research on PTSD and CVD is the under-representation of women and non-veteran populations. The majority of studies to date have focused on military veterans, particularly men, due to the high prevalence of combat-related PTSD in this group and the availability of large, well-characterised cohorts such as the U.S. Department of Veterans Affairs databases [9]. While these studies have been instrumental in elucidating the link between PTSD and CVD, they may not fully capture the biological, behavioural, and social variability present in civilian populations.

Women are consistently under-represented in PTSD-CVD research, despite evidence suggesting that they are twice as likely as men to develop PTSD following trauma exposure [75]. Moreover, women tend to experience different trauma types, including sexual violence, domestic abuse, and childbirth-related trauma, which may have distinct psychophysiological and cardiovascular consequences compared to combat or disaster-related trauma more common among men [76]. Data from the Nurses’ Health Study II demonstrated that women with high PTSD symptom burden had a significantly increased risk of developing myocardial infarction and stroke, even after adjusting for conventional cardiovascular risk factors [10]. These findings underscore the importance of expanding research beyond veteran and male-dominant samples.

Non-veteran civilian populations are also under-represented in large-scale longitudinal research. Civilian trauma often co-occurs with socioeconomic disadvantage, limited healthcare access, and chronic stressors such as discrimination or community violence, all of which may influence both PTSD trajectories and cardiovascular outcomes [77].

Consequently, findings from veteran-based studies may not generalise to broader populations. Furthermore, most studies are conducted in high-income countries, limiting understanding of PTSD and CVD in low- and middle-income regions where trauma exposure and healthcare disparities are widespread [6].

Knowledge gaps and future research

Need for Mechanistic Studies

Although substantial epidemiological evidence links PTSD with elevated CVD risk, important gaps remain in understanding the underlying biological mechanisms. Most current findings are based on cross-sectional or short-term studies of autonomic dysfunction, inflammation, endothelial impairment, and neuroendocrine abnormalities. Longitudinal mechanistic studies that track biological changes over time following trauma exposure are needed to clarify temporal pathways and identify modifiable physiological targets. Reviews of existing research emphasise that mechanistic data remain fragmented and that more integrative, multi-system investigations are required to better understand how PTSD contributes to cardiovascular deterioration [14].

Lack of Randomized Trials Assessing Whether PTSD Treatment Reduces CVD Incidence

Another major knowledge gap is the absence of randomized controlled trials evaluating whether effective PTSD treatment can reduce subsequent CVD incidence. Observational studies suggest that improvement in PTSD symptoms is associated with better cardiometabolic profiles, including reductions in blood pressure, inflammatory markers, and autonomic dysregulation [21]. However, randomised trials are essential to determine causality and assess whether psychological therapies, pharmacological treatments, or combined interventions can directly influence long-term cardiovascular outcomes. Experts in the field have noted that such trials represent a crucial next step for translating mechanistic insights into preventive strategies [21].

More Data Required from Low- and Middle-Income Countries

Most research on PTSD and CVD has been conducted in high-income countries, despite the high burden of trauma exposure and limited mental health resources in low- and middle-income countries. Global epidemiological reviews highlight wide disparities in trauma prevalence and major gaps in population-based data from regions affected by conflict, displacement, and socioeconomic hardship [4]. More inclusive research is needed to understand how cultural factors, healthcare access, and regional differences in trauma exposure influence the association between PTSD and CVD.

Role of Social Determinants and Cumulative Trauma Exposure

Social determinants of health, including SES, discrimination, neighbourhood environment, and access to care, play an important role in shaping both PTSD risk and cardiovascular vulnerability. Studies have demonstrated that individuals with lower SES or chronic exposure to social adversity are more likely to develop PTSD and experience higher rates of cardiometabolic disease [8]. In addition, recent work suggests that cumulative trauma exposure, rather than single-event trauma, may have a stronger and more persistent effect on both PTSD severity and cardiovascular risk. Greater attention to these structural and contextual factors is needed to develop targeted interventions and equitable public health responses.

Sex- and Age-Specific Analyses

Emerging evidence indicates that the relationship between PTSD and CVD is modified by both sex and age, although the patterns differ depending on the type of cardiovascular outcome and the presence of comorbidities.

Several cohort studies have examined whether the PTSD-CVD link differs by sex. For example, a large civilian cohort study found that PTSD was associated with incident myocardial infarction and stroke in both men and women with broadly similar effect sizes [18]. Another large U.S. Army cohort of predominantly younger servicemembers found that associations of PTSD with hypertension were significantly stronger among men than among women [2]. Taken together, these data suggest that while PTSD elevates cardiovascular risk in both men and women, the magnitude or mediating pathways of that risk may differ by sex.

Age likewise appears to modify the PTSD-CVD association, particularly for intermediate-risk phenotypes such as hypertension. In the large Army cohort cited above, younger age (< 40 years) was associated with a stronger PTSD-hypertension association compared with older age (≥ 40 years) [78]. Another registry-based Korean study found that the elevation in risk of coronary artery disease and ischaemic stroke associated with PTSD was more prominent in younger individuals [12]. These findings raise the possibility of a “window of susceptibility” whereby mid-life or younger exposure to PTSD may carry heightened cardiovascular risk, possibly because of concurrent accumulation of other cardiovascular risk factors.

The sex and age heterogeneity of PTSD-related cardiovascular risk has several important implications. First, future longitudinal studies should routinely report sex- and age-stratified results and formally test for interaction terms. Second, sex-specific biological mechanisms (for example, variations in autonomic reactivity, hormonal milieu, or vascular ageing) and age-related changes (for instance, arterial stiffness, endothelial senescence, and metabolic accumulation) warrant detailed mechanistic study. Third, in clinical settings, screening for cardiovascular risk in patients with PTSD may need to incorporate stratified risk adjustment: for example, younger men with PTSD and no other metabolic comorbidities may still harbour elevated risk for hypertension, calling for early prevention. Finally, interventions to mitigate cardiovascular sequelae of PTSD might need tailoring by sex and age; what works in middle-aged men may not translate identically to younger women or to older adults.

Limitations

In addition to the limitations inherent in the existing scientific literature, several constraints of this manuscript should be acknowledged. This article is structured as a narrative review rather than a systematic review or meta-analysis, and therefore, the evidence selection process did not follow predetermined inclusion criteria or formal risk-of-bias scoring. Because of this, selection bias and publication bias cannot be excluded. The manuscript also does not provide quantitative synthesis or pooled effect estimates, meaning that the associations described between PTSD and CVD rely on qualitative assessment and cannot be interpreted as precise measures of risk. Furthermore, although evidence from multiple regions was incorporated, the body of research available is still largely dominated by studies conducted in high-income settings. As a result, the generalisability of conclusions to trauma-exposed populations in low- and middle-income regions is uncertain.

Finally, PTSD frequently coexists with depression, anxiety, and substance use disorders, and this review could not fully separate the cardiovascular effects directly attributable to PTSD from those linked to overlapping psychiatric and behavioural pathways. These considerations highlight the need for future research using standardised diagnostic approaches, expanded international populations, mechanistic profiling, and interventional study designs to strengthen causal understanding and improve clinical application.

## Conclusions

PTSD is a significant but often overlooked contributor to CVD. Across epidemiological studies, PTSD is consistently associated with higher risks of hypertension, metabolic syndrome, coronary artery disease, stroke, and cardiovascular mortality. These findings are supported by mechanistic evidence demonstrating that PTSD promotes autonomic imbalance, HPA axis dysregulation, inflammation, endothelial dysfunction, and adverse health behaviours that jointly accelerate cardiometabolic decline.

Despite this evidence, PTSD is rarely incorporated into cardiovascular risk assessment or routinely screened for in clinical practice, even though trauma disproportionately affects vulnerable populations. The bidirectional relationship, where cardiac events can also trigger PTSD, further underscores the need for integrated, trauma-informed care. Future research should include longitudinal mechanistic studies, randomised trials evaluating whether PTSD treatment reduces CVD risk, and expanded investigation in low- and middle-income countries. Addressing PTSD within cardiovascular prevention frameworks may meaningfully improve long-term outcomes for trauma-exposed individuals.
